# Engineering Muscle Networks in 3D Gelatin Methacryloyl Hydrogels: Influence of Mechanical Stiffness and Geometrical Confinement

**DOI:** 10.3389/fbioe.2017.00022

**Published:** 2017-04-07

**Authors:** Marco Costantini, Stefano Testa, Ersilia Fornetti, Andrea Barbetta, Marcella Trombetta, Stefano Maria Cannata, Cesare Gargioli, Alberto Rainer

**Affiliations:** ^1^Department of Engineering, Università Campus Bio-Medico di Roma, Rome, Italy; ^2^Department of Biology, Tor Vergata Rome University, Rome, Italy; ^3^Department of Chemistry, Sapienza University of Rome, Rome, Italy

**Keywords:** gelatin methacryloyl, hydrogel stiffness, C2C12 differentiation, geometrical confinement, skeletal muscle

## Abstract

In this work, the influence of mechanical stiffness and geometrical confinement on the 3D culture of myoblast-laden gelatin methacryloyl (GelMA) photo-crosslinkable hydrogels was evaluated in terms of *in vitro* myogenesis. We formulated a set of cell-laden GelMA hydrogels with a compressive modulus in the range 1 ÷ 17 kPa, obtained by varying GelMA concentration and degree of cross-linking. C2C12 myoblasts were chosen as the cell model to investigate the supportiveness of different GelMA hydrogels toward myotube formation up to 2 weeks. Results showed that the hydrogels with a stiffness in the range 1 ÷ 3 kPa provided enhanced support to C2C12 differentiation in terms of myotube number, rate of formation, and space distribution. Finally, we studied the influence of geometrical confinement on myotube orientation by confining cells within thin hydrogel slabs having different cross sections: (i) 2,000 μm × 2,000 μm, (ii) 1,000 μm × 1,000 μm, and (iii) 500 μm × 500 μm. The obtained results showed that by reducing the cross section, i.e., by increasing the level of confinement—myotubes were more closely packed and formed aligned myostructures that better mimicked the native morphology of skeletal muscle.

## Introduction

Skeletal muscle (SM) is a highly dynamic and plastic tissue able to modify its intrinsic size or strength following electric impulse, mechanical loading, or diet. SM accounts for about 30–45% of body weight, being the most abundant among human body tissues (Buckingham and Montarras, 2008). This tissue can self-repair relatively small damages resulting from tears, small lacerations, strains, or toxins *via* a three-stage process that involves demolition, repair, and remodeling of myofibers. However, SM tissue cannot restore significant tissue loss that can arise after severe trauma, invasive surgeries, degenerative diseases, or simply as a consequence of aging (Tidball, 2011; Milner and Cameron, 2013).

Tissue engineering holds great promise for the fabrication of artificial muscles to be used for *in vitro* studies and for the replacement of diseased or injured muscle tissue (Bach et al., 2004; Levenberg et al., 2005). However, due to its structural complexity, engineering a functional muscle tissue *in vitro* still represents a daunting task. Two of the most challenging aspects consist in attaining (i) a proper 3D organization of myotubes into highly packed and aligned structures (as to mimic the native SM tissue) and (ii) an advanced maturation of the myotubes in terms of formation and development of sarcomeres. To address these challenges, different strategies have been developed in the recent past (Almany and Seliktar, 2005; Fuoco et al., 2012, 2015; Manabe et al., 2012; Melchels et al., 2012; Malda et al., 2013; Juhas et al., 2014; Heher et al., 2015; Madden et al., 2015; Kang et al., 2016; Morimoto et al., 2016). In particular, to promote a proper 3D organization of myotubes that could mirror the natural organization of muscle fascicles, bioreactors have been designed to stimulate the constructs loaded with myogenic progenitors either mechanically or electrically (Powell et al., 2002; Manabe et al., 2012; Ito et al., 2014; Heher et al., 2015; Kang et al., 2016). These works demonstrated the possibility of obtaining highly oriented myofibers: for example, mouse myoblast cell line C2C12 cultured under static strain showed an increase in myotube alignment and sarcomere maturation (Heher et al., 2015); the same cell line, when electrically stimulated, showed an increase in the maturation of myoblasts, with a percentage of contractile myotubes as high as 80% (Manabe et al., 2012). However, all the presented approaches showed limitations in terms of process scalability. On the other hand, to better understand the process of myogenesis and sarcomerogenesis, researchers have primarily studied the influence of substrate stiffness on the spreading, elongation, and cooperative fusion of myoblasts (Engler et al., 2004; Gilbert et al., 2010). In these studies, substrate stiffness has been demonstrated not only to affect the formation of syncytia, but also to play a key role in myotube maturation and in the assembly of the sarcomeric unit.

Although successful in determining an optimal stiffness value for the maturation of myotubes (~12 kPa), these studies have been performed by seeding cells directly on the surface of the biomaterials (2D substrates). This may represent a bias for all the studies in which cells are encapsulated within hydrogels (3D substrates) experiencing an actual 3D environment.

In this study, we investigate the influence of two parameters—namely, hydrogel stiffness and geometrical confinement—on the *in vitro* differentiation of C2C12 myoblasts encapsulated in gelatin methacryloyl (GelMA) hydrogels. First, we formulated a set of precursor hydrogel solutions with increasing GelMA concentrations and we tuned the stiffness of the resulting hydrogels by varying the degree of UV-induced cross-linking. After a thorough mechanical characterization, we used those formulations for the preparation of cell-laden hydrogels in combination with C2C12 murine myoblasts and, at desired time points, we qualitatively evaluated the development of myotube structures by means of bright-field and fluorescence microscopy. Finally, we set up a robust and facile method to fabricate string-like cell-laden hydrogel structures with different cross sections to study the effect of such geometrical confinement on the degree of alignment of the resulting myotubes.

## Materials and Methods

### Synthesis of GelMA

**Figure 1:**
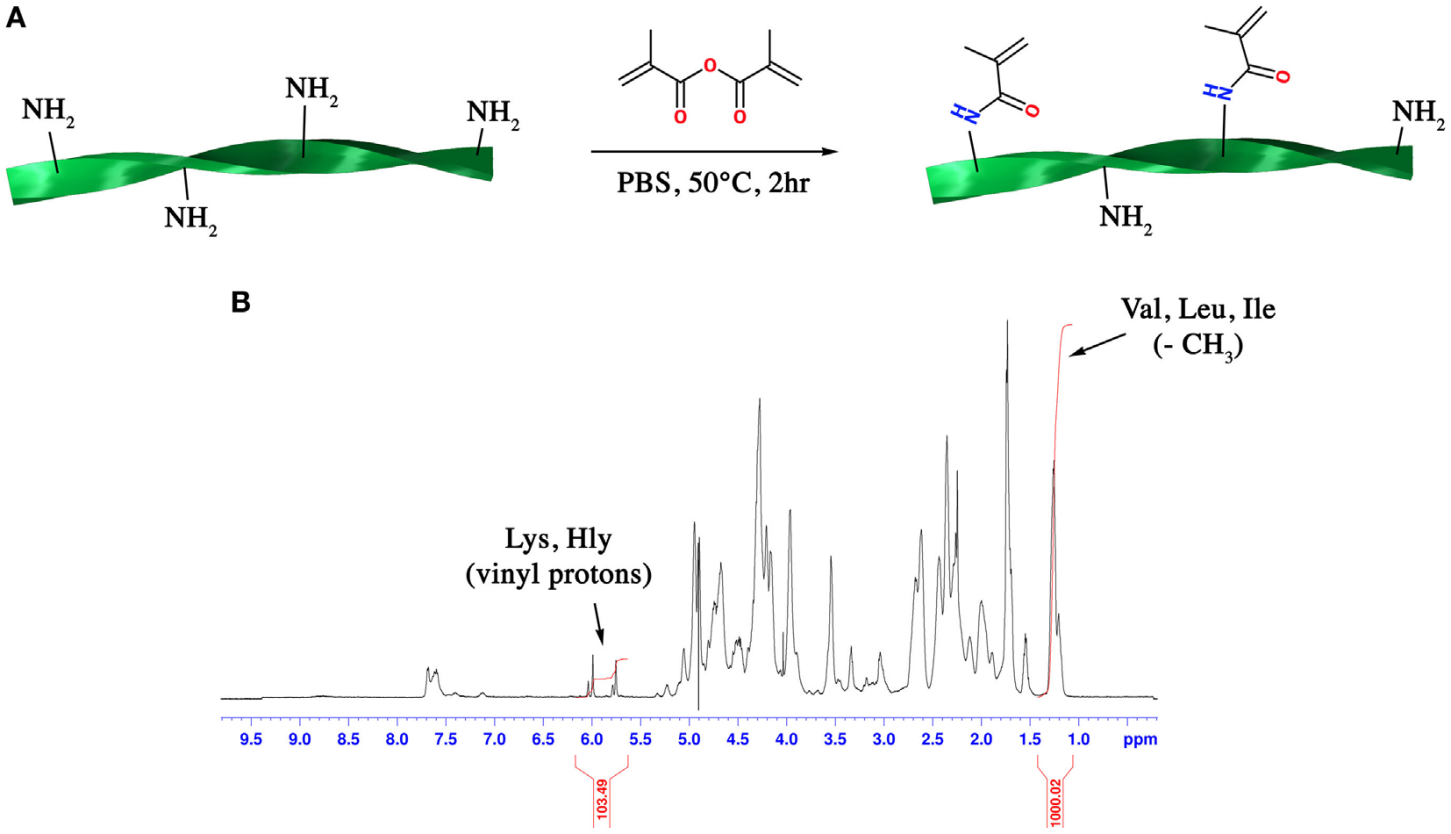
**(A)** Scheme of the synthetic route for the synthesis of gelatin methacryloyl (GelMA) and **(B)** ^1^H-NMR spectrum of GelMA in which the peaks relative to hydrophobic alkyl side chains of valine (Val), leucine (Leu), and isoleucine (Ile) and methacrylamide substituent groups of lysine (Lys) and hydroxylysine (Hly) amino acids are shown.

Gelatin methacryloyl was synthesized following a previously published protocol (Costantini et al., 2016). Briefly, gelatin (type A3, ~300 Bloom from porcine skin) was dissolved at 10% (w/v) in PBS at 60°C. Methacrylic anhydride (MA, 0.08 mL per gram of gelatin) was then added to the gelatin solution dropwise under vigorous stirring and the mixture was allowed to react for 2 h (Figure 1A). After a five-fold dilution with additional warm PBS, the GelMA solution was dialyzed against deionized water using 12–14 kDa cutoff dialysis tubes (Spectrum Laboratories) for 6 days at 50°C to remove unreacted MA and additional by-products. GelMA was lyophilized and stored at −20°C until use.

### Determination of the Degree of Substitution (DS)

^1^H-NMR (Bruker AVANCE AQS 600 MHz) operating at 600.13 MHz was used to determine the DS of the synthesized GelMA according to a previously published work (Ovsianikov et al., 2011). Briefly, GelMA was dissolved in warm D_2_O, and the ^1^H-NMR spectrum was acquired when the sample reached an equilibrium temperature of 40°C. The DS could be calculated by applying the following formula:

$$\text{DS}\left(\%\right)={\text{Mol}}_{\text{Val},\text{Leu},\text{Ile}}\times \frac{I_{5.7\text{ppm}}}{I_{1.2\text{ppm}}}\times \frac{100}{{\text{Mol}}_{\text{Lys},\text{Hly}}}$$

where Mol_Val,Leu,Ile_ is the total number of moles of valine, leucine, and isoleucine amino acids present in gelatin; Mol_Lys,Hly_ is the total number of moles of lysine and hydroxylysine present in gelatin; *I*_1.2 ppm_ is the integration of the peak ascribed to the resonance of hydrophobic alkyl side chains of valine, leucine and isoleucine while *I*_5.7 ppm_ is the integration of the peak ascribed to the vinyl proton of methacrylamide substituent groups. On the basis of the known amino acid composition of gelatin, the total number of moles for each amino acid could be calculated.

### Fabrication of Soft Mold in Polydimethylsiloxane (PDMS) Elastomer

Soft molds were fabricated in PDMS by replica molding of a PMMA (Plexiglas) master produced by CNC milling. Briefly, we designed the model of the mold with a CAD software (AutoCAD, Autodesk). The mold was composed of three microchannels with the same length (*L* = 16.5 mm) but different cross sections: 2,000 μm × 2,000 μm, 1,000 μm × 1,000 μm, and 500 μm × 500 μm. To enhance the mechanical stability and to facilitate the handling of the hydrogels, we added an additional *U*-shaped channel as to create a frame to the three microchannels. Then, we imported the 3D model in a CAM software (SolidCAM, Dassault Systèmes) and generated a tool path code to obtain a positive master, which was precision-milled out of a 5-mm-thick Plexiglas sheet using a CNC milling machine (Proxxon MF70 with Mach3 software, Newfangled Solutions). Finally, PDMS (Sylgard 184, Dow Corning, 10:1 pre-polymer to catalyst ratio) was poured on top of the Plexiglas master, degassed, and cross-linked at 60°C for 3 h. Prior to use, PDMS molds were sterilized by thorough washing in pure ethanol (99.8%) followed by washing in sterile PBS.

### Preparation of Hydrogels and *In Vitro* Culture

#### Influence of Hydrogel Stiffness on Myoblast Differentiation

To study the influence of hydrogel stiffness on myoblast differentiation, we prepared four GelMA solutions with a polymer concentration of 3, 4, 6, and 8% w/v in PBS, respectively. 1 mg/mL Irgacure 2959 (BASF) was added to the solutions as a radical photoinitiator. Solutions were then laden with C2C12 myoblasts at a concentration of 2 × 10^7^ cells/mL. The cell suspensions were finally cast into cylindrical molds (diameter = 4 mm, height = 2 mm) and cross-linked by low-dose UV irradiation (365 nm, 1.3 mW/cm^2^ for 4 min). The obtained hydrogels were cultured *in vitro* for 14 days in DMEM (Gibco) supplemented with 10% heat-inactivated fetal bovine serum, 100 IU/mL penicillin, and 100 mg/mL streptomycin at 37°C and 5% CO_2_ humid atmosphere. For each of the formulated GelMA solutions, experiments were performed in triplicate.

#### Influence of Geometrical Confinement on Myotube Orientation

To study the influence of geometrical confinement on C2C12 differentiation, we prepared hydrogels with different cross section using the aforementioned PDMS mold. GelMA concentration was set at 4% w/v, as this value resulted the best environment for myoblast differentiation from the first propaedeutic series of experiments. To draw accurate statistics of myotube alignment within the hydrogel structures, experiments were performed in triplicate. Cell density, cross-linking time, and *in vitro* culture conditions were the same as in the previous experiments.

### Mechanical Testing

Gels were characterized in terms of their compressive stiffness under unconfined compression using an Instron 3365 universal tester (Norwood, MA, USA). Cylindrical specimens (8 mm in diameter, 4 mm in height) of formulated GelMA were tested at room temperature (RT) up to 50% final strain with the following parameters: 0.01 N preload force, 0.125/min strain rate. Values for the compressive modulus were calculated from the initial linear region (0–20% of strain) of the obtained stress–strain curves. Each measurement was performed in triplicate and results are reported as the mean ± SD.

### Immunofluorescence (IF)

Cells encapsulated within GelMA hydrogels were fixed in PFA 2% and processed for fluorescence immunocytochemistry as previously described (Scardigli et al., 2008). Briefly, samples were re-hydrated with PBS and cells were permeabilized with 0.2% v/v Triton X-100 in PBS for 30 min. Subsequently, samples were incubated with mouse anti-myosin heavy chain (MHC, Clone MF20 DSHB, 1:2) primary antibody diluted in blocking buffer (0.2% Triton X-100 and 20% heat-inactivated goat serum in PBS) for 20 min at RT. After several washes with buffer, sections were incubated with anti-mouse FITC (Chemicon, 1:500) secondary antibody. Samples were counterstained with DAPI to detect nuclei, washed three times with wash buffer, and mounted on Vectashield antifade mounting medium (Vector Laboratories). Samples were imaged with a Nikon Eclipse 2000-TE microscope equipped with a CoolSNAP MYO CCD camera (Photometrix) and MetaMorph software.

### Image Analysis

Image analysis was performed using ImageJ software. The distributions of myotube orientation within the hydrogel structures following *in vitro* culture was obtained by analyzing IF micrographs (MHC signal) with OrientationJ plugin (Püspöki et al., 2016). The plugin evaluates the orientation for every pixel of the image based on the structure tensor and provides an orientation distribution plot as an output.

## Results

### Influence of Hydrogel Stiffness on Myoblast Differentiation

One of the main features that influences the network density and stiffness of GelMA hydrogels is the DS of ε-amino groups of lysine and hydroxylysine with methacrylic moieties. Despite the complexity of the ^1^H-NMR spectrum of GelMA, an accurate and quantitative determination of DS can be carried out through a ^1^H-NMR-based relative quantitation method. This method can be easily applied to GelMA since its amino acid composition is well known (Eastoe, 1955). The peak around 1.2 ppm (Figure 1B) can be ascribed to the resonance of the hydrophobic alkyl side chains of valine (Val), leucine (Leu), and isoleucine (Ile). These hydrophobic side chains can be considered chemically inert and thus they do not participate in the reaction with MA. On the other hand, the peaks at 5.7 and 6.0 ppm can be ascribed to the vinyl proton of methacrylamide substituent groups of lysine and hydroxylysine amino acids. By using these two peaks as reference peaks and by applying the following formula (see Section “Materials and Methods” for more details):

$$\begin{array}{l} \text{DS}\left(\%\right)={\text{Mol}}_{\text{Val},\text{Leu},\text{Ile}}\times \frac{I_{5.7\text{ppm}}}{I_{1.2\text{ppm}}}\times \frac{100}{{\text{Mol}}_{\text{Lys},\text{Hly}}}\\ \;=0.384\times \frac{103.49/2}{1000.02}\times \frac{100}{0.0385}=51.6\% \end{array}$$

a DS of 51.6% for the synthesized GelMA was obtained.

**Figure 2:**
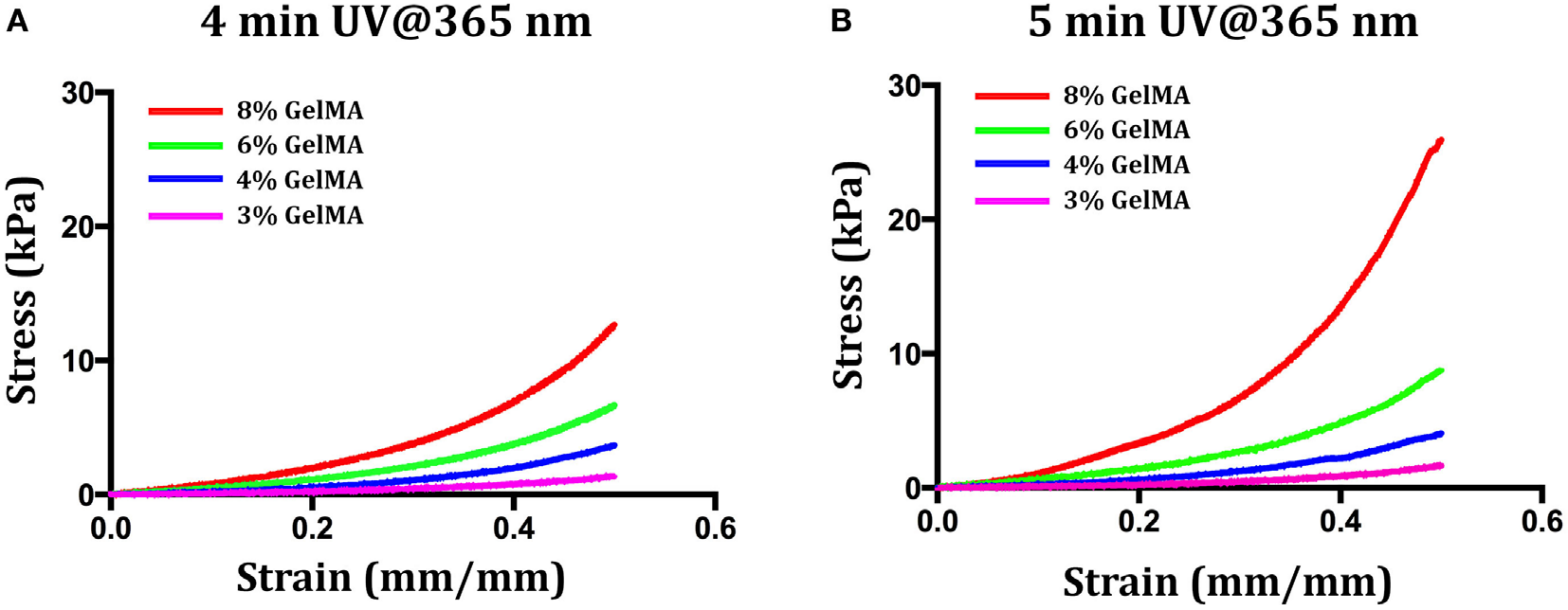
**Stress–strain curves for different values of gelatin methacryloyl (GelMA) concentration (3, 4, 6, and 8% w/v) and UV cross-linking time [4 min (A) and 5 min (B)]**.

Following its synthesis, purification and characterization, GelMA was used for the preparation of a set of hydrogels. Since our first goal was to evaluate the influence of hydrogel stiffness on myoblast differentiation, before encapsulating the myogenic precursor, we explored two different variables that influence hydrogel stiffness: (i) the concentration of GelMA in the precursor hydrogel solutions and (ii) the UV crosslinking time. First, we performed few experiments to determine the lowest limits for the two parameters—i.e., the minimum concentration and the shortest UV cross-linking time that would eventually give rise to a gel. It turned out that for concentrations below 3% w/v—independently of the UV-light cross-linking time—it was not possible to obtain a stable hydrogel. On the contrary, for a GelMA concentration of 3% w/v, it was possible to obtain stable hydrogels for a minimum cross-linking time of 4 min (UV dose ≈ 310 mJ/cm^2^). These values of GelMA concentration and UV light cross-linking time were thus taken as lowest levels for the two parameters. Then, we prepared other three solutions with GelMA concentrations up to 8% w/v—namely 4, 6, and 8% w/v. The four GelMA solutions were UV cross-linked for either 4 or 5 min, and the resulting hydrogels were then mechanically tested to determine their compressive behavior. The stress–strain plots for the formulated hydrogels are reported in Figure 2, while Table 1 summarizes the values of compressive modulus calculated from the stress–strain data. As it can be noticed, both GelMA concentration and UV cross-linking time have an impact on the compressive modulus, which increases with increasing either of the two parameters. Since it is well known that myoblasts, when cultured on top of hydrogel systems, sense the substrate stiffness and, as demonstrated in previously published works (Engler et al., 2004), undergo a better differentiation on substrates having a stiffness around 8 ÷ 12 kPa, we decided not to considerably exceed this range and thus we did not prepare hydrogels with GelMA concentration higher than 8% w/v. Furthermore, since the effect of UV irradiation time for a given composition (% GelMA) was minor, we decided to limit cell experiments to the 4-min irradiation group, as to keep the UV dose to cells as low as possible.

**Table 1:** **Values of the compressive modulus of elasticity as calculated from stress–strain curves for hydrogels at different levels of gelatin methacryloyl (GelMA) concentration and UV dose**.

GelMA (% w/v)	Elastic modulus (kPa)	Elastic modulus (kPa)

	4 min UV@365 nm	5 min UV@365 nm
3	1.06 ± 0.07	1.19 ± 0.12
4	2.45 ± 0.05	3.20 ± 0.10
6	5.81 ± 0.10	8.74 ± 0.22
8	10.04 ± 0.13	16.55 ± 0.80

**Figure 3:**
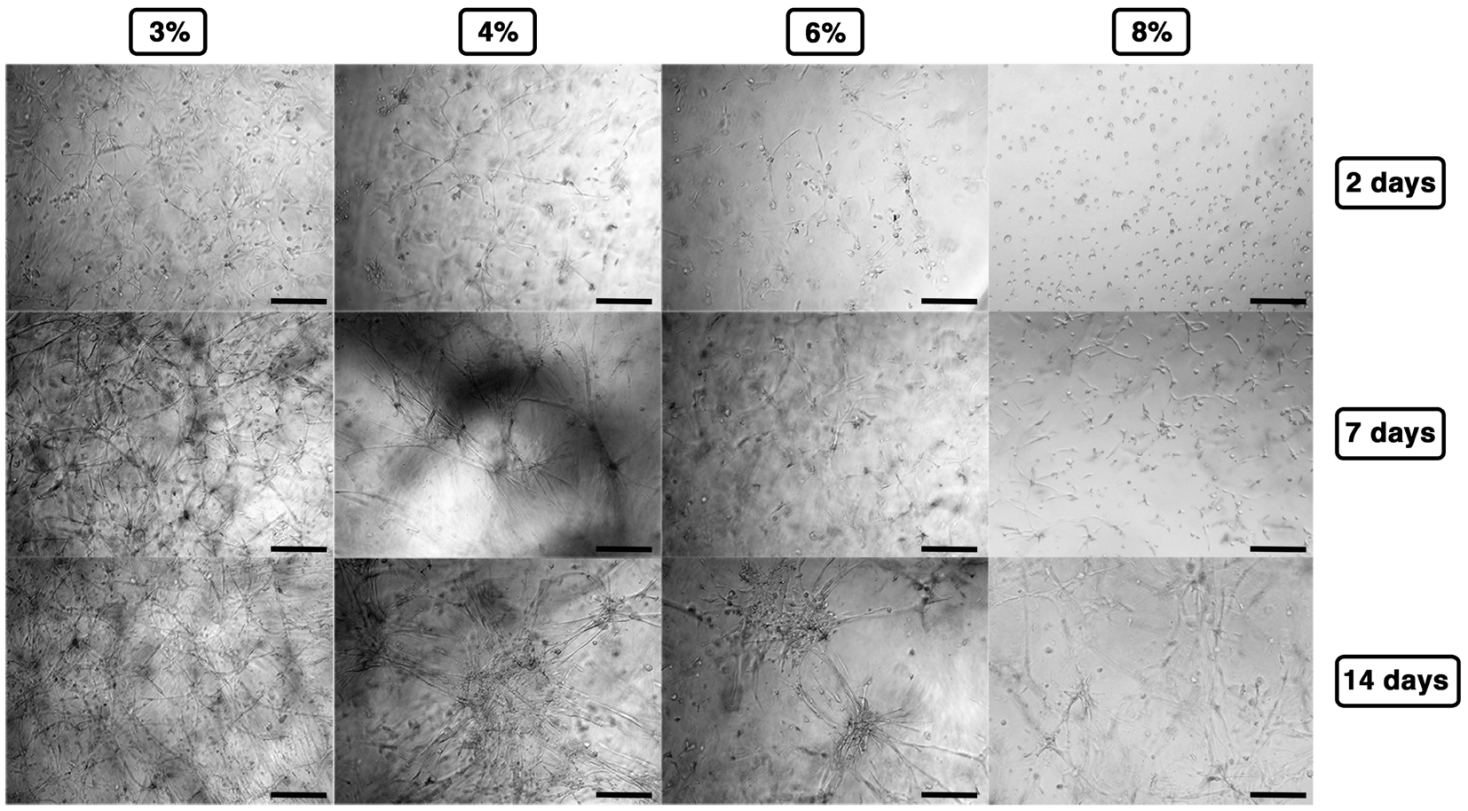
**Gelatin methacryloyl stiffness affecting C2C12 myogenesis as observed by phase contrast microscopy up to 14 days**. Scale bars: 100 µm.

Cell-laden hydrogels were timely observed by phase contrast microscopy up to 14 days, and the results are summarized in Figure 3. At lower concentrations (3–4% w/v), C2C12 rapidly spread within the hydrogel matrices revealing the formation of short myotubes already 48 h after polymerization, whereas at higher concentrations, cells presented limited spreading (6% w/v) or even almost exclusively round shape (8% w/v). At later time points—7/14 days after polymerization—C2C12 encapsulated into 3% GelMA hydrogel displayed a remarkable amount of myotubes forming a 3D-entangled network homogeneously distributed in the hydrogel volume. Similarly, 4% GelMA hydrogel supported a pronounced formation of myotubes, comparable to the one obtained with 3% GelMA, albeit with a slightly less homogeneous distribution. On the contrary, at higher GelMA concentrations (6–8%), C2C12 presented a remarkable detriment in myogenic differentiation, with minor myotube formation generally localized in clusters.

### Influence of Geometrical Confinement on Myotube Orientation

**Figure 4:**
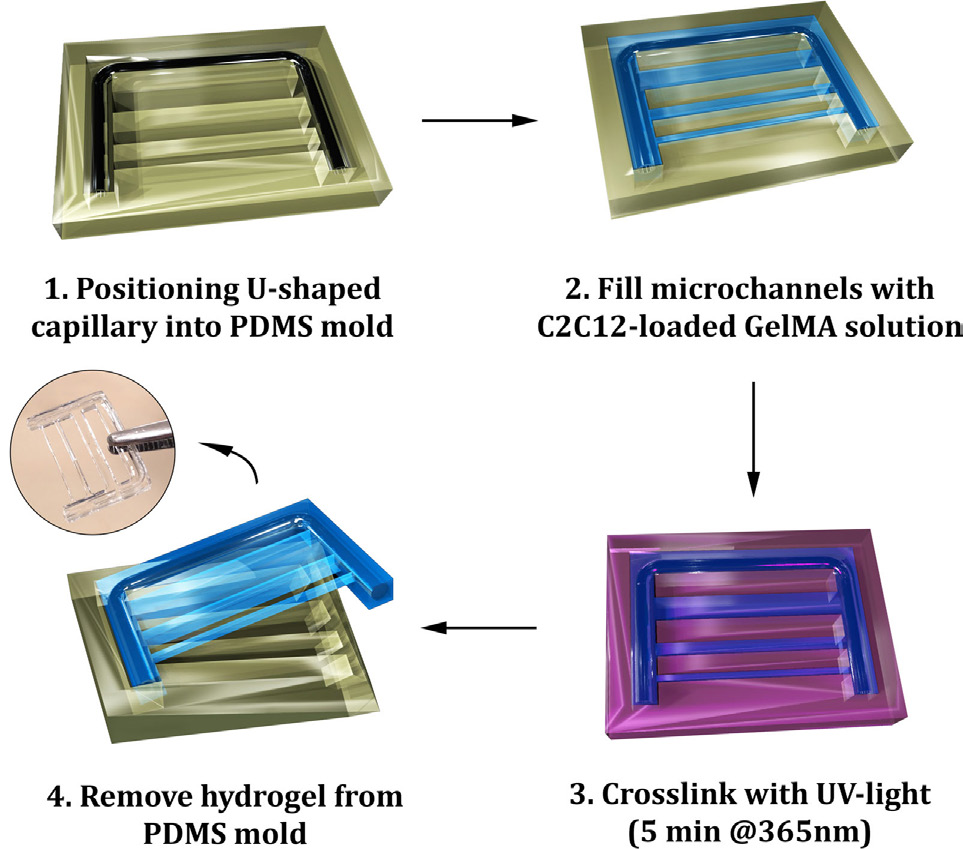
**Scheme of the micromolding process for the fabrication of gelatin methacryloyl (GelMA) hydrogels: a *U*-shaped glass capillary is embedded within the hydrogel to act as a supporting frame for the hydrogel strings having different cross sections**.

The first propaedeutic experiment evidenced a clear correlation between matrix stiffness and the ability of myoblasts to undergo efficiently myogenesis. Results showed that the best candidates were those at lower GelMA concentration (3 and 4%)—i.e., with lower compressive moduli. Despite the performances of 3% GelMA were slightly better, we decided to use 4% GelMA as these hydrogels were easier to handle. After determining the most suitable GelMA concentration for myogenic differentiation, a micromolding approach was established to investigate myotube organization under different confinement regimes, as sketched in Figure 4. The mold was designed to produce cell-laden thin hydrogel strings at different cross sections—namely 2,000 μm × 2,000 μm, 1,000 μm × 1,000 μm, and 500 μm × 500 μm—connected to a *U*-shaped supporting frame, further reinforced by a modeled glass capillary at its core. This solution endowed the structure with enhanced rigidity to support the ensuing myostructures and restraining hydrogel contraction due to myotube tensions.

**Figure 5:**
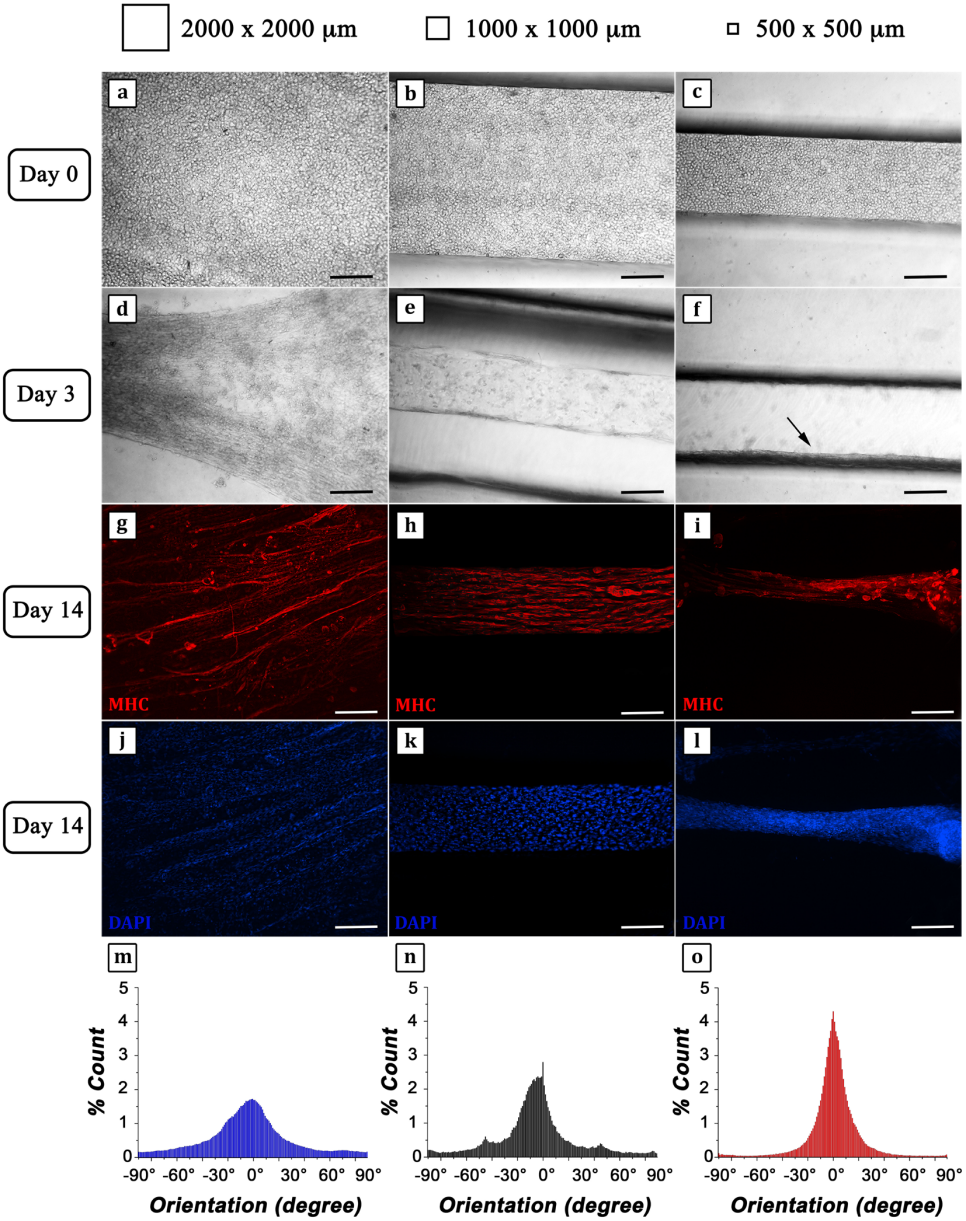
**C2C12 cells encapsulated into 4% GelMA at different degrees of geometrical confinement**. **(A–F)** Phase contrast micrographs of C2C12 captured 24 h **(A–C)** and 72 h **(D–F)** after cross-linking. From left to right: 2,000 μm × 2,000 μm; 1,000 μm × 1,000 μm; 500 μm × 500 μm. Black arrow in **(F)** indicates myostructure shrinkage occurring in the thinner structure. **(G–L)** Immunofluorescence (IF) against myosin heavy chain (MHC) (red) **(G–I)** following 14 days culture; nuclei were counterstained by DAPI (blue) **(J–L)**. Scale bars: 100 µm. **(M–O)** Myotube orientation distribution plots (with 0° corresponding to the direction of the major axis of symmetry of each hydrogel structure) calculated from IF micrographs (MHC signal).

C2C12 cells were successfully retained within the three different hydrogel strings as confirmed by phase contrast micrographs showing a regular round shape (Figures 5A–C). After 3 days of culture, the structures revealed a remarkable compaction (Figures 5D–F) most likely due to C2C12 differentiation and myotube formation, particularly highlighted in the hydrogel with the smallest cross section (arrow in Figure 5F). Fourteen days after cell encapsulation, IF analyses were performed to evaluate the organization of MHC positive myotubes (Figures 5G–L). Micrographs showed a considerable parallel orientation for the myostructure cultured in the smallest (500 μm × 500 μm) and middle (1,000 μm × 1,000 μm) hydrogel structures (Figures 5H,I), while in the largest ones (2,000 μm × 2,000 μm), parallel organization was just partially achieved (Figure 5G), revealing the significant importance of proper geometrical confinement of myogenic precursors during the SM differentiation process. This can be better appreciated from the myotube orientation distribution plots (Figures 5M–O). In all cases, myotube orientation distribution was peaked along the hydrogel string axis. However, by reducing the hydrogel cross section, the distribution plots appeared increasingly sharp (Figures 5N,O), confirming that the geometrical confinement plays a significant role in myotube alignment.

## Discussion

Despite the notable achievements and technological progress of the last decade, assembling a functional artificial SM is still a daunting task. Numerous approaches have been developed and tested with the aim of recapitulating the SM architectures and a partial degree of biomimicry, especially in terms of myotube orientation, has been achieved (Ito et al., 2014; Fuoco et al., 2015; Heher et al., 2015; Madden et al., 2015).

Pioneering studies have demonstrated that myogenic precursors are particularly sensitive to the stiffness of the substrate on top of which they are cultured, demonstrating that sarcomerogenesis is favored only for a small range of stiffness values (~10 ÷ 14 kPa) (Engler et al., 2004). Although representing significant proofs of concept, these studies suffer from two main disadvantages: (i) the employed strategies are not scalable and (ii) they poorly resemble the native 3D conditions in which SM tissue develops.

These values—obtained for 2D cell culture—should be carefully reviewed as myogenic precursors are often encapsulated within hydrogel systems and, in this condition, they sense an actual 3D environment. From the cellular perspective, 2D and 3D culture conditions represent two distinct scenarios and, hence, the response of myogenic precursors might be completely different.

Given these premises, we tried to evaluate the influence of hydrogel stiffness and of geometrical confinement on the *in vitro* generation of engineered muscle networks within a hydrogel system. To this aim, we encapsulated C2C12 cells within GelMA hydrogels with a stiffness in the range ~1 ÷ 10 kPa.

Interestingly, we found that, as the stiffness of the matrix increases, the ability of C2C12 to undergo rapid and efficient, myogenesis highly decreases, and the best results were obtained for the hydrogels with the lowest stiffness (~1 ÷ 3 kPa, GelMA 3 ÷ 4% w/v). These results are in contrast with those published in literature for 2D culture of C2C12. A thorough explanation of this phenomenon is not simple. Most likely, we can speculate that C2C12—when cultured either in 2D or 3D—experience different focal adhesion configurations that influence cell polarization and cell signaling pathways (Wozniak et al., 2004; Eriksson et al., 2007).

Furthermore, to undergo myogenesis, C2C12 cultured in 3D need to partially digest the surrounding matrix to fuse with adjacent cells and, reasonably, they need to activate metabolic pathways for matrix metalloproteinases. This could partially explain why C2C12 did not differentiate significantly in the stiffest hydrogels due to high matrix density. Additionally, the diffusion rate of metabolites and wastes is inversely proportional to the matrix stiffness, and this might further hamper C2C12 differentiation within the stiffest hydrogels (Drury and Mooney, 2003).

Finally, we investigated the influence of geometrical confinement on myogenic precursors embedded in 4% GelMA. Results showed a remarkable influence of the hydrogel cross section on the architectural organization of differentiated myotubes. In all cases, we observed a rapid compaction of hydrogels with a significant decrease in their cross sections. This phenomenon is mediated by cells: in fact, as cell fusion proceeds, the arising myotubes produce a tension that is capable to shrink the gels. Furthermore, IF analysis revealed that the degree of myotube organization was influenced by hydrogel cross sections. In particular, we noticed that myotube distribution was more homogeneous in the hydrogel strings with smaller cross sections (1,000 μm × 1,000 μm and 500 μm × 500 μm), in which a more physiological 3D organization with the formation of highly aligned bundles could also be observed.

In conclusion, we have demonstrated that both hydrogel stiffness and geometrical confinement play a crucial role in the differentiation of myogenic precursors in a 3D culture environment. However, a thorough understanding of these phenomena is still missing, and further investigations are required to better clarify how they can affect the generation of functional SM tissue *in vitro*.

## Author Contributions

MC, CG, and AR designed the experiments; MC, ST, EF, AB, and CG carried out experiments; AB performed ^1^H-NMR GelMA characterization; CG, SC, MT, and AR supervised the project. MC, AB, CG, MT, and AR wrote the manuscript. All co-authors contributed to discussion and analysis of the data.

## Conflict of Interest Statement

The authors declare that the research was conducted in the absence of any commercial or financial relationships that could be construed as a potential conflict of interest.
